# Acute and chronic effects of plyometric exercise performed with hypoxia on post-activation performance enhancement (PAPE)

**DOI:** 10.1371/journal.pone.0335247

**Published:** 2025-10-27

**Authors:** Betul Coskun, Michael J. Hamlin

**Affiliations:** 1 Faculty of Sport Sciences, Erciyes University, Kayseri, Türkiye; 2 Department of Tourism, Sport and Society, Lincoln University, Christchurch, New Zealand; Università degli Studi di Milano: Universita degli Studi di Milano, ITALY

## Abstract

In the literature, no study is available either to investigate the effects of conditioning activity (CA) applied in hypoxic conditions on post-activation performance enhancement (PAPE) or to examine whether hypoxic long-term training can affect PAPE. This study aims to test the effects of plyometric exercise applied under hypoxia on PAPE, which is the acute effect; and to test the same effect again after an 8-week plyometric training, which is a potential chronic effect on the acute performance improvement after an adaptation with training. Nineteen team-sports athletes received 8-week drop-jump (DJ) training in Low-Normobaric Hypoxia (Low-NH, n = 8), Normobaric-Normoxia (NN, n = 6), or High-Normobaric Hypoxia (High-NH, n = 5) conditions (SpO_2_ of 90%, 97–100%, and 80%, respectively) two times per week. PAPE was tested at the 2^nd^ and 4^th^ minutes of recovery after normoxic and hypoxic CA with 1x5 DJs at the pre-test, and tested after an 8-week training period following a normoxic and hypoxic CA with 1x8 DJs at the post-test. As a result of repeated measures ANOVA to identify the acute effects, only under normoxic conditions, DJ-height was significantly higher in the 2^nd^ (31.7 ± 4.9 cm) and 4^th^ minute (31.6 ± 4.3 cm) than baseline (30.1 ± 4.7 cm) (p < 0.05). Regarding the chronic-effect results, only the High-NH group significantly increased DJ-height from baseline (31.6 ± 4.5 cm) to the 2^nd^ (33.7 ± 5.9 cm) and 4^th^ minutes (34.5 ± 4.6 cm) (p < 0.05), without testing condition (hypoxic/normoxic) separately, at the end of the 8-week training period. It is concluded that plyometrics with acute hypoxic CA have no beneficial effect on PAPE responses, but 8 weeks of plyometric training with normobaric hypoxia may lead to an adaptation to induce improved PAPE.

## Introduction

Both acute [1–3] and long-term [4–6] exercise under hypoxic conditions are considered valuable methods to boost sea-level performance. Another method for improving acute performance is post-activation performance enhancement (PAPE), which refers to the transient increase in performance that occurs after completing a short conditioning activity that acts to enhance athletic performance [7,8]. There is a scarcity of research to investigate the PAPE response following an acute exercise with hypoxia and following a long-term training adaptation with hypoxia.

Post-activation performance enhancement is known as a physiological phenomenon that induces a short-term enhancement in power and strength performance through previous muscle activation [9]. It is thought that muscle fatigue and potentiation coexist shortly after exercise, and the net balance between this fatigue and potentiation determines the subsequent performance. If the exercise is not too stressful and there is sufficient rest, potentiation occurs; however, if the exercise is overly stressful or inadequate rest is given, the participant’s coping mechanisms will be stressed and fatigue will ensue [9–11].

Post-activation potentiation is a neuromuscular phenomenon characterized by an increase in myosin light chain phosphorylation, especially in type II muscle fibers, after a high-intensity muscle activity, which increases force production in the first few minutes after contraction. It is a muscle contraction response after an intense voluntary contraction. Besides, PAPE refers to the voluntary performance increase observed during the longer rest periods compared to post-activation potentiation, in addition to mechanisms such as myosin phosphorylation after a high-intensity conditioning activity. Proposed physiological mechanisms behind the beneficial effect of PAPE include an increase in myosin light chain phosphorylation, especially in type II muscle fibers, increased muscle temperature, alteration in intramuscular water content, and a change in coordination or motivation [12].

It is well known that exercise under hypoxic conditions creates an increased metabolic stress [13] with a lower overall workload [14]. This increased stress is thought to be due to the increased burden placed on Type II muscle fibers under hypoxic exercise conditions [15]. It is also known that the most critical factors for PAPE to occur are the volume and intensity of the conditioning activity and the rest period between the conditioning activity and subsequent athletic performance [16]. We hypothesize that the additional stress caused by the low oxygen content during hypoxia will increase the metabolic stress of the conditioning activity [17] without an increase in the physical stress. Generating greater metabolic stress without physical stress may exacerbate the potentiation without increasing fatigue in the PAPE equation, thereby improving athletic performance. However, just what hypoxic level is appropriate for this type of activity is unclear [1,18], along with what recovery period is best to drive the greatest potentiation in performance.

Therefore, this study aimed to examine the effects of plyometric exercise applied under hypoxia acutely on PAPE over several recovery times. We also aimed to test the differences in the PAPE response after an 8-week plyometric training period with high and low hypoxia to investigate whether hypoxic plyometric training improved PAPE.

## Method

### Participants

Using G*Power (G*Power 3.1.9.7) with a medium effect size, an alpha level of 0.05, and a power (1-beta) of 0.80, a total of 21 participants was required as a priori sample size for this study. In total, we started with 24 team-sport athletes (such as basketball, netball, rugby, hockey, and cricket), however, due to missing training or testing sessions, we finished the study with 19 athletes (19.0 ± 1.1 years, 178.4 ± 6.5 cm, 79.8 ± 13.0 kg), consisting of 9 females (age 19.1 ± 1.1 years; body weight 73.2 ± 10.5 kg; body height 175.2 ± 6.8 cm) and 10 males (age 18.9 ± 1.2 years; body weight 85.7 ± 12.6 kg; body height 181.3 ± 4.8 cm).

To ensure that each group had a similar distribution of baseline performance to reduce any potential confounding effects, we randomly matched participants on their pre-test DJ performances first and then accordingly divided them into three groups with eight people per group. However, at the end of the study, we completed the research with a lower number of participants in the training groups: Low-Normobaric Hypoxia (Low-NH, n = 8) (19.0 ± 1.3 years, 178.6 ± 8.4 cm, pre: 80.2 ± 16.5 kg, post: 80.8 ± 17.1 kg), Normobaric Normoxia (NN, n = 6) (19.2 ± 1.2 years, 178.8 ± 4.2 cm, pre: 76.5 ± 10.3 kg, post: 76.7 ± 11.1 kg), and High-Normobaric Hypoxia (High-NH, n = 5) (18.8 ± 1.0 years, 177.6 ± 6.6 cm, pre: 83.3 ± 11.0 kg, post: 84.9 ± 11.9 kg). Participants who had experienced lower extremity surgery or a musculoskeletal injury within the past 2 years [19] or who had used performance-enhancing drugs [20] were not included in the study. After explaining the research, written informed consent was taken from the participants, and Lincoln University Human Ethics Committee approved this study (HEC2023−26).

### Experimental procedure

The experimental research design in this study was a pretest-posttest group design. To investigate the acute effect of plyometric exercise applied under hypoxic conditions on PAPE, we had the athletes complete DJ exercises under normoxic and hypoxic conditions (on different days) as the conditioning activity. We then used DJ tests during the recovery period (2^nd^ and 4^th^ min.) to witness any PAPE change.

To investigate the chronic effect, we used the same athletes but split them into 3 training groups (Low-NH, NN, and High-NH) to train for 8 weeks. We performed the same testing protocol after the 8-week training period. We measured participants’ PAPE after the conditioning activity in normoxia and the conditioning activity in hypoxia as the post-test. We applied a counterbalanced crossover design to test PAPE under two conditions (hypoxia and normoxia). To minimize any potential learning or order effects, half of the participants performed the tests first in the hypoxic condition, followed by in the normoxic condition, while the other half completed the tests in the reverse order. Thus, we made an effort to enhance internal validity by distributing any potential practice effects across conditions.

### Hypoxic and normoxic breathing

**Fig 1:**
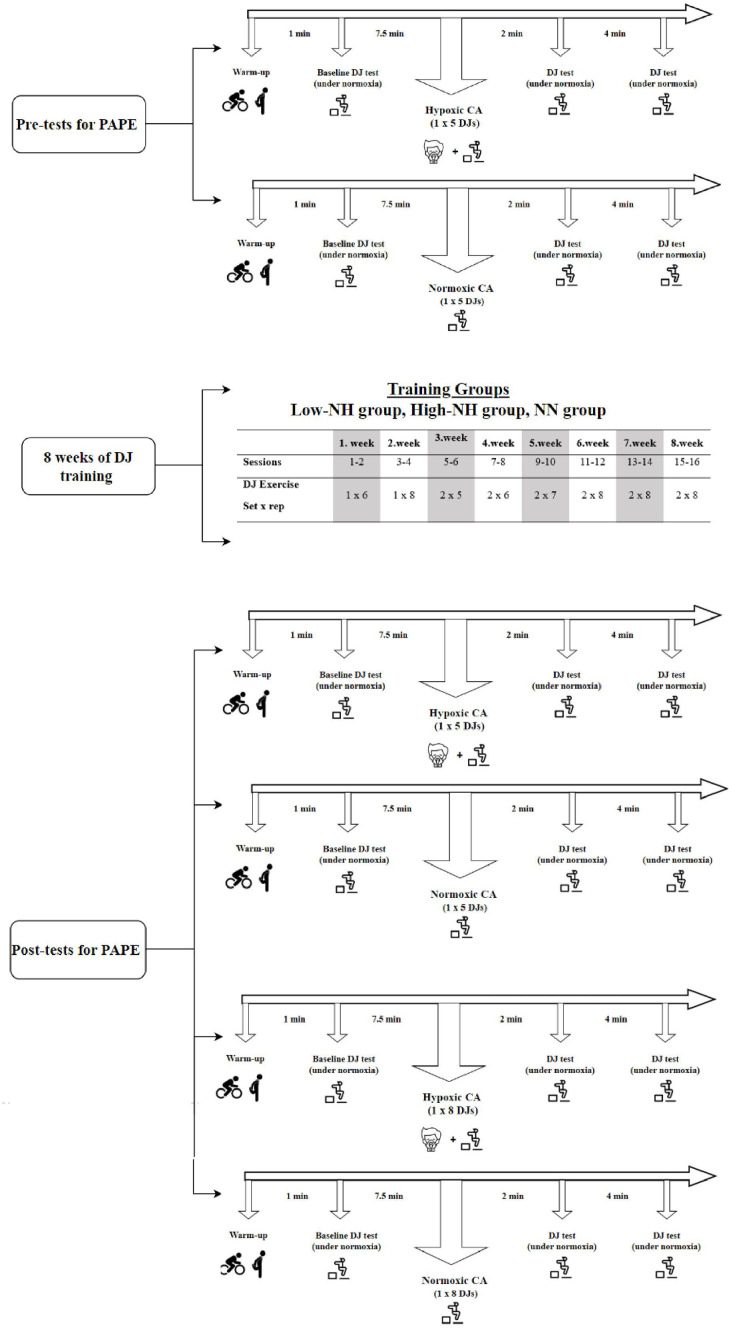
Representation of the experimental design.

Normobaric hypoxia was supplied by a hypoxicator (GO2Altitude hypoxicator, Biomedtech, Victoria, Australia) using a fully automated biofeedback control system that automatically adjusts the fractional inspired concentration of oxygen (F_I_O_2_) to sustain a given SpO_2_ level. Therefore, we set SpO_2_ levels instead of altitude levels. While it is clear that oxygen saturation falls with increasing altitude, it is not entirely certain to determine a specific threshold value that would be considered the “normal” saturation level at a given altitude [21]. Since there is no certain saturation level at a particular elevation, we used results of the previous studies to determine the expected values of SpO_2_ for approximately 2500 m [22,23], 4500 m [24,25] hypoxia levels, and sea level by reviewing their oxygen saturation data. The hypoxicator was set by the biofeedback control system to sustain an SpO_2_ of 90% for the Low-NH group, 97–100% for the NN group, and 80% for the High-NH group. Athletes performed plyometric training 2 days per week for 8 weeks under these three different conditions. They were instructed to refrain from plyometric exercises and maintain their regular training routines during the study. Fig 1 provides a summary of the experimental design in this study.

### DJ training protocol

The training program was generated by considering previous study findings on DJ performance [20,26–28].

To avoid overloading the athletes, who already had heavy training schedules, we designed a training programme to start with a relatively low volume over the first 2 weeks and build up slowly during the next 6 weeks. Additionally, we maintained a low repetition rate during exercises (Table 1) to target the phosphagen pathways as recommended by Sandler [29].

**Table 1:** The 8-week training protocol.

Week	1^st^ week	2^nd^ week	3^rd^ week	4^th^ week	5^th ^week	6^th^ week	7^th^ week	8^th^ week
**Sessions**	1-2	3-4	5-6	7-8	9-10	11-12	13-14	15-16
**DJ Exercise**								
**Set x rep**	1 x 6	1 x 8	2 x 5	2 x 6	2 x 7	2 x 8	2 x 8	2 x 8
**Total Jump/week**	12	16	20	24	28	32	32	32
**Total**	For each experimental group, 196 DJs							

Each training session began with a 10-minute warm-up, which consisted of low-intensity cycling for 5 minutes followed by 5 minutes of stretching exercises, followed by a few submaximal vertical jumps. To adapt to hypoxia, participants wore hypoxia masks before the warm-up session (immediately before cycling) and continued being exposed to hypoxia during stretching, plyometric exercises, and 5-minute cool-down sections [6]. During exercise, oxygen saturation and heart rate were observed to ensure the participants were effectively exposed to hypoxia. Instruction was given to perform the exercises with maximum effort and explosively. A rest period of 15 seconds between repetitions and 2 minutes between sets was applied [27,28]. During the jumps, participants were asked to place their hands on their hips and extend one leg straight before dropping on both legs to the ground and jumping quickly and as high as possible while executing DJ. We instructed participants to minimize the contact time with the ground and maximize the jump height [26]. These instructions for DJ execution were supplied during both training sessions and testing sessions.

### PAPE test protocol

Initially, a standardized warm-up protocol was executed, which contained 5 minutes of low-intensity cycling and 5 minutes of dynamic stretching focusing on the major muscles of the lower limb [30]. After the warm-up, baseline tests were obtained via two trials of DJ from a height of 40 cm.

As CA, we used 1x5 DJs. Since it is a new topic, we found only the study of Ramos-Campo, Malta [17], related to the PAPE effect influenced by hypoxia. The researchers applied 4x5 horizontal jumps as a CA [17]. However, according to our laboratory experiences for another research, we observed that increased fatigue in exercise, with the addition of hypoxia to the plyometric exercises, has the potential to impair performance. Accordingly, we decreased the number of exercises and included only DJ in this current study. On the other hand, the study of Chen, Lo (30) showed advances as a result of 1x5 DJ exercise in normoxic conditions, too.

**Fig 2:**
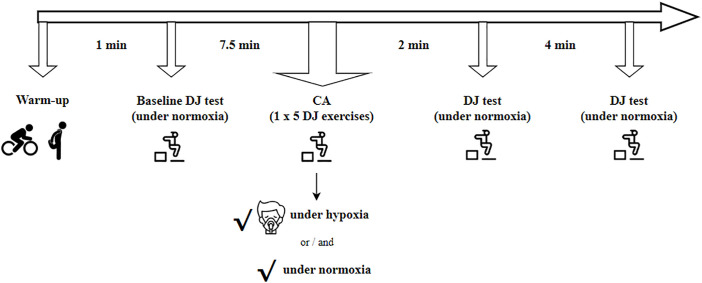
Representation of the test design for PAPE.

For hypoxic CA, after 7.5 minutes of passive rest with the SpO_2_ of 90% [17], 1x5 DJ exercises were performed with 5-second rest [30] in this study. The same protocol was performed with 7.5 minutes of passive rest in normoxic conditions, as well as for normoxic CA (Fig 2). Seitz and Haff [31] stated that the post-activation potentiation effect can be accomplished earlier following a plyometric CA compared to traditional (high/moderate intensity) CAs and recommended the first 0.3–4 minutes for the highest post-activation potentiation effect seen after a plyometric CA. Additionally, in the only study conducted on systemic hypoxia, performance improvement was detected after 3 minutes of passive rest following jump exercises as a form of rewarming [17]. Therefore, in this study, DJ tests were performed at two time points during rest, at the 2^nd^ and 4^th^ minutes, following a five-repetition DJ exercise set as the PAPE protocol (Fig 2). HR (heart rate), oxygen saturation, and RPE (rating of perceived exertion) (Borg’s 6–20 scale) were estimated at these time points. Testing PAPE responses, under either hypoxia or normoxia conditions, was conducted on a separate day at least 24 hours apart in a random order, before and after the 8-week training period.

We decided to test PAPE at 2 time points, but we also preferred trained individuals as participants in our study against the risk of an extra potentiation or accumulated fatigue effect induced by the 2^nd^ time point, which may be seen at the 4^th^ minute. The rest interval between the 2^nd^ and 4^th^ minutes of testing time can mitigate fatigue, since the participants were trained athletes in this study. Tillin and Bishop [32] reported that fatigue decreases more rapidly than post-activation potentiation during the recovery period after CA, and also trained individuals with a more fast-twitch fiber distribution may be more likely to benefit from post-activation potentiation compared to individuals who do not. Seitz and Haff [31] also support this by stating that stronger people can perform a greater post-activation potentiation effect than the weaker individuals, and also, they can develop fatigue resistance. Moreover, to reveal an optimal potentiation, multiple sets were recommended instead of single sets, especially for trained individuals [33].

It is not possible to achieve continuous improvement with long-term training applied at the same rate, but higher levels of muscle conditioning can be achieved by appropriately varying program variables such as number of sets and repetitions and length of rest period. Also, it is known that most of the strength gains occur in the first 4–8 weeks [34]. Therefore, at the pre-tests, we tested PAPE with 1x5 DJs as CA, but at the post-tests, we also tested with 1x8 DJs, too.

All DJ tests were measured with SmartJump Portable Jump Mat (Vald Performance Pty Ltd, Brisbane, Australia), and the variables of contact time (CT), flight time (FT), jump height, reactive strength index (RSI), and power given automatically by the jump mat were evaluated in our study.

### Statistical analysis

We used mean ± SD as descriptive data and the Shapiro-Wilk test as a normality test before using ANOVAs. To examine the acute PAPE effect, a two-way ANOVA with repeated measures was used (condition: hypoxia and normoxia, testing time: baseline, 2^nd^ min., and 4^th^ min.). As for the chronic PAPE effect, DJ performance variables were analyzed with mixed-design repeated measures ANOVA (3x2x2x3) to test the effects of group (NN, High-NH, Low-NH), condition (hypoxia and normoxia), time (pre- and post-training period), and testing time (baseline, 2^nd^ min., and 4^th^ min.). Significance level was accepted at <0.05. All statistical analyses were performed by the Statistical Package for Social Sciences (version 29) (SPSS Inc., Chicago, IL, USA). Evaluations with the smallest worthwhile change (SWC) have been recommended for studies based on exercise performance analyses because of the small sample size or the typical error of measurements. For athletes with high levels of fitness, it was suggested that the standard deviation be multiplied by 0.2 to determine SWC [35]. We also calculated the smallest worthwhile change (SWC) for significant DJ heights through the standard deviation (SD) of all baseline DJ heights, which was multiplied by 0.2 [35,36].

## Results

**Fig 3:**
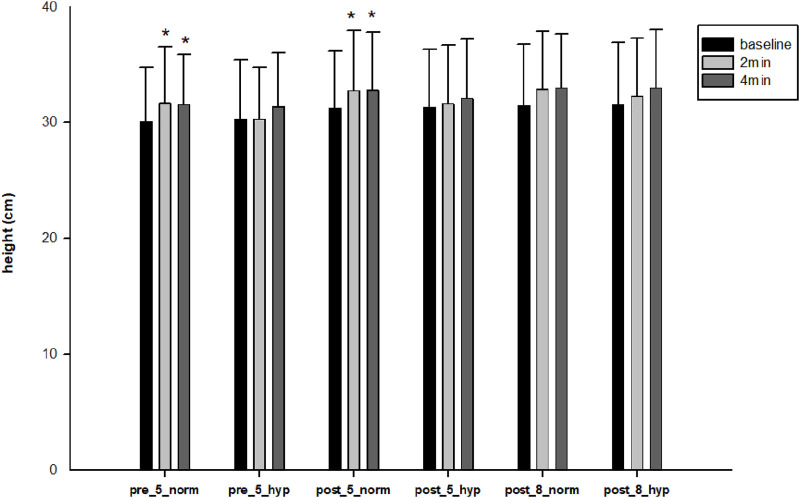
Test time points differences in terms of two conditions of hypoxia and normoxia for pre-test with 5 DJs (acute effect), and post-training period with 5- and 8-DJs. The mean ± standard deviation for jump height at baseline, 2 min., and 4 min. after normoxic and hypoxic DJ exercise.* Significantly different from the baseline of the same condition (p < 0.05). **pre_5_norm **= pre-test with 5 DJs under normoxia, **pre_5_hyp **= pre-test with 5 DJs under hypoxia, **post_5_norm **= post-test with 5 DJs under normoxia, **post_5_hyp **= post-test with 5 DJs under hypoxia, **post_8_norm **= post-test with 8 DJs under normoxia, **post_8_hyp** = post-test with 8 DJs under hypoxia.

Because of the significant interaction between condition and test, pairwise comparisons were performed with Bonferroni adjustment. Only after the normoxic CA, jump height significantly increased at the 2^nd^ (p = 0.015) and at 4^th^ minutes (p = 0.043) compared to baseline (Fig 3), along with power at the 2^nd^ (p = 0.015) and 4^th^ minutes (p = 0.046) compared to baseline (Table 2).

**Table 2:** Repeated ANOVA results of pre-test for the acute effect of hypoxic and normoxic CA on PAPE (n = 19).

Variable	After normoxic CA			After hypoxic CA			ANOVA *p*		
	Baseline	2 min	4 min	Baseline	2 min	4 min	Condition	Test	Condition x Test
**Height (cm)**	**30.1 ± 4.7**	31.7 ± 4.9*	31.6 ± 4.3*	30.3 ± 5.1	30.3 ± 4.4	31.4 ± 4.6	0.129	**0.013**	**0.044**
**RSI (m/s)**	1.25 ± 0.26	1.32 ± 0.27	1.27 ± 0.26	1.25 ± 0.26	1.24 ± 0.26	1.31 ± 0.24	0.611	0.172	0.050
**CT (ms)**	248.2 ± 55.2	247.3 ± 54.1	254.2 ± 53.4	249.9 ± 54.9	252.1 ± 55.4	247.6 ± 54.0	0.997	0.850	0.467
**FT (ms)**	485.3 ± 56.2	506.6 ± 39.7	506.4 ± 34.6	495.3 ± 43.0	496.0 ± 36.2	504.9 ± 37.9	0.874	**0.023**	0.090
**Power (W)**	**2943.6 ± 281.6**	3037.6 ± 297.5*	3033.0 ± 258.3*	2956.0 ± 308.7	2956.4 ± 269.4	3024.0 ± 282.0	0.146	**0.014**	**0.047**
**HR (bpm)**	97.8 ± 10.7	96.8 ± 13.8	95.7 ± 14.3	96.1 ± 17.2	92.1 ± 15.4	91.5 ± 14.7	0.132	0.208	0.666
**S**_**P**_**O**_**2**_ **(%)**	97.7 ± 0.5	97.9 ± 0.7	98.0 ± 0.7	98.0 ± 0.6	97.7 ± 0.9	97.9 ± 0.7	0.947	0.476	0.293
**RPE**	9.8 ± 2.3	10.4 ± 2.2	10.4 ± 2.3	9.7 ± 2.3	10.3 ± 2.2	10.4 ± 2.2	0.713	**0.002**	0.852

Significant statistical differences are indicated in bold for repeated ANOVA results.

*Significantly different from the baseline of the same condition (p < 0.05). Height, jump height; RSI, reactive strength index; CT, contact time; FT, flight time; HR, heart rate; RPE, rating of perceived exertion.

**Fig 4:**
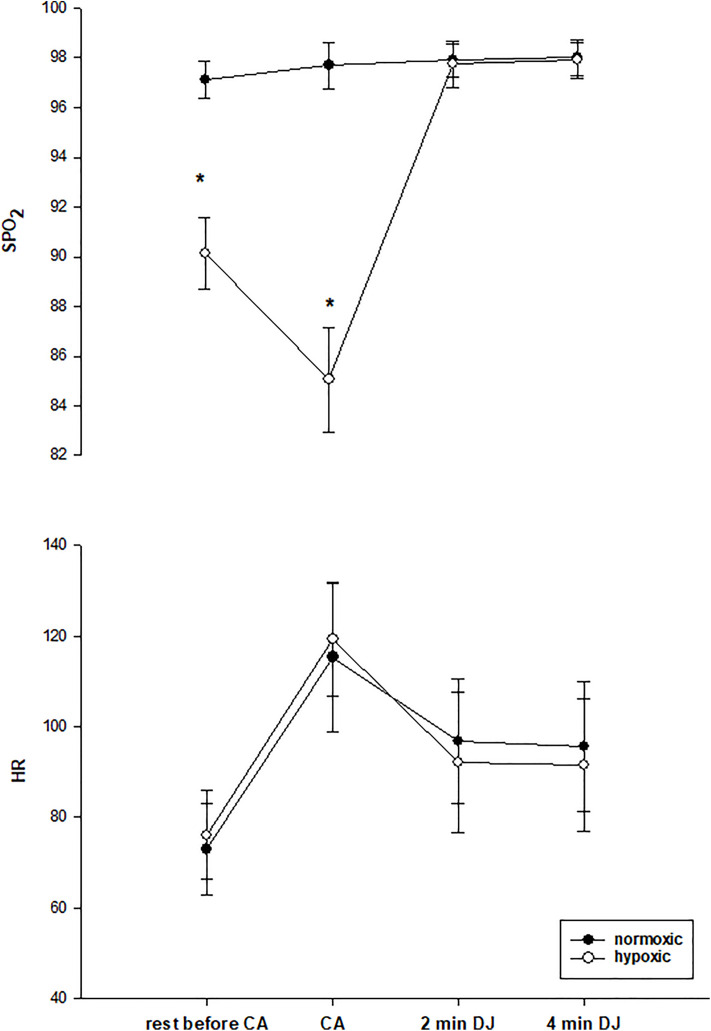
For acute effect evaluations, S_P_O_2_ (A) and heart rate (B) changes during rest under the CA condition, immediately after CA (1x5 DJS), and DJ tests at two time points. * Significantly different from other conditions (p < 0.05).

The changes of training variables, S_P_O_2_ and heart rate, during rest under the CA condition, immediately after CA (1x5 DJs), and DJ tests at two time points are given in Fig 4.

For FT and RPE, there was a significant main effect of testing time, meaning significant differences among testing time points (baseline, 2^nd,^ and 4^th^ min.) (Table 2). Pairwise comparisons showed that FT (505.7 ms) was significantly higher than the baseline FT (490.3 ms, p = 0.047) in the 4^th^ minute of recovery. As for RPE, baseline RPE (9.8) was significantly lower than both the 2^nd^ minute RPE (10.3, p = 0.009) and the 4^th^ minute RPE (10.4, p = 0.017) without the condition effect (hypoxic or normoxic condition).

As for the 5-repetition test protocol results, interaction between condition and testing time was significant (p = 0.003, partial η^2^ = 0.30). Only normoxic conditions showed overall (without pre- and post-test difference) significant increases in jump height at the 2^nd^ (32.6 cm, p = 0.002) and 4^th^ minutes (32.7 cm, p < 0.001) compared to the baseline (31.06 cm). Considering pre- and post-test 5-repetition test results separately, again only normoxic conditions revealed significantly higher jump heights at the 2^nd^ and 4^th^ minutes than baseline (p < 0.05) (Fig 3).

For the 8-repetition test protocol results, interaction was significant between testing time and group (p = 0.048, partial η^2^ = 0.25), therefore, we continued with pairwise comparisons and found that only the High-NH training group increased jump height significantly at the 2^nd^ minute (31.9 cm, p = 0.019) and 4^th^ minute (32.9 cm, p < 0.001) compared to baseline (30.0 cm) and also from 2^nd^ minute to 4^th^ minute (p = 0.05), without time (pre-test and post-test) factor difference. However, pairwise comparisons with time factor differences (for pre- and post-test separately) were also presented in Table 3.

**Table 3:** Results for the overall PAPE response without the condition difference of CA.

Variable	Group	Pre-training			Post-training with 5-rep. CA			Post-training with 8-rep. CA		
		Baseline	2 min.	4 min.	Baseline	2 min.	4 min.	Baseline	2 min.	4 min.
**Height** **(cm)**	**NN (n = 6)**	31.1 ± 3.1	31.7 ± 4.2	32.1 ± 4.2	31.5 ± 3.0	32.2 ± 4.4	31.8 ± 4.0	32.1 ± 4.0	33.4 ± 3.7	33.2 ± 3.9
	**High-NH (n = 5)**	**28.4 ± 4.9**	30.0 ± 3.4	31.2 ± 4.3*	31.4 ± 4.1	32.4 ± 4.4	32.7 ± 5.3	**31.6 ± 4.5**	33.7 ± 5.9*	34.5 ± 4.6*
	**Low-NH (n = 8)**	30.7 ± 6.0	31.1 ± 5.7	31.1 ± 4.9	33.7 ± 7.7	34.9 ± 8.1	35.0 ± 8.1	34.1 ± 8.6d	35.0 ± 7.6	35.4 ± 8.0d

Significant statistical differences are indicated in bold.

*Significantly different from the baseline of the same testing period (pre/post) for the same training group (p < 0.05).

For RSI, only the High-NH group showed significantly higher RSI at 2^nd^ (1.40, p = 0.015) and 4^th^ minutes (1.40, p = 0.007) compared to baseline (1.27), for the 5-rep. test protocol.

As for CT, no significant differences were observed in CT among testing time points, between training groups, or between CA conditions (p > 0.05).

For the variable of FT, while we found significantly higher FT at the 2^nd^ (514.3 ms, p = 0.002) and 4^th^ minutes (514.8 ms, p < 0.001) than baseline (496.3 ms) only after the normoxic CA for the 5-rep. protocol, we did not find any significant condition difference at any test time points for the 8-rep. test protocol (p > 0.05). Only the High-NH group presented a significant difference at the 2^nd^ (503.6 ms, p = 0.010) and 4^th^ minutes (509.7 ms, p < 0.001) compared to the baseline (484.4 ms) for the 5-rep. test protocol. Similarly, for the 8-rep. protocol, only the High-NH group showed a significant difference at the 2^nd^ (508.3 ms, p = 0.003) and 4^th^ minutes (517.1 ms, p < 0.001) compared to baseline (485.3 ms) and between the 2^nd^ and 4^th^ minutes (p = 0.047) as well.

As for the power, only the normoxic condition showed higher power results at the 2^nd^ (3096.8 W, p = 0.002) and 4^th^ minutes (3100.4 W, p < 0.001) than baseline (3003.1W) with the 5-rep. test protocol. Only the High-NH group showed significantly different power results at the 2^nd^ (3050.6 W, p = 0.022) and 4^th^ minutes (3115.9 W, p < 0.001) compared to baseline (2941.7 W) and also between the 2^nd^ and 4^th^ minutes (p = 0.041), with the 8-rep. test protocol.

For RPE, with the 5-rep. test, only the Low-NH group showed higher RPE at the 2^nd^ (9.5, p = 0.003) and 4^th^ minutes (9.8, p = 0.001) than baseline (8.1) after hypoxic CA, and at the 2^nd^ minute (9.5, p = 0.034) compared to baseline (8.9) after normoxic CA at pre-test. As for the post-test, the Low-NH group presented higher RPE at the 2^nd^ (9.1, p = 0.023) and 4^th^ (9.3, p = 0.05) minutes than baseline (8.3) only after normoxic CA. With the 8-rep. test protocol, only the Low-NH group presented higher RPE at the 2^nd^ (9.3, p < 0.001) and 4^th^ minutes (9.4, p = 0.002) than baseline (8.5) without CA condition difference.

**Fig 5:**
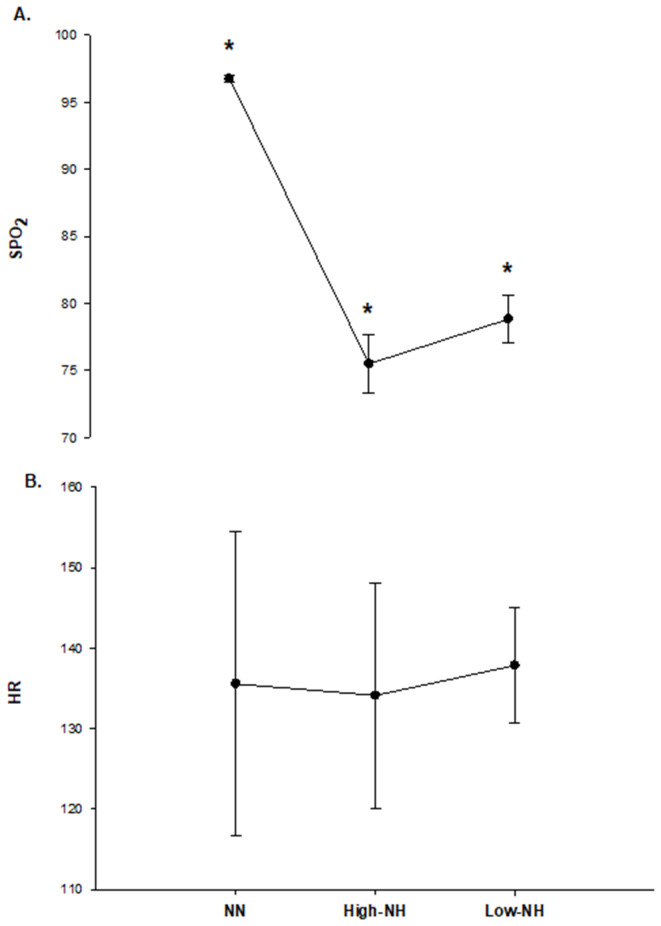
S_P_O_2_ (A) and heart rate (B) mean values of DJ exercises during eighteen training sessions for each training group. *Significantly different from other training groups (p < 0.05).

Training variables, S_P_O_2_ and heart rate, mean values of DJ exercises during eighteen training sessions for each training group were presented in Fig 5.

**Fig 6:**
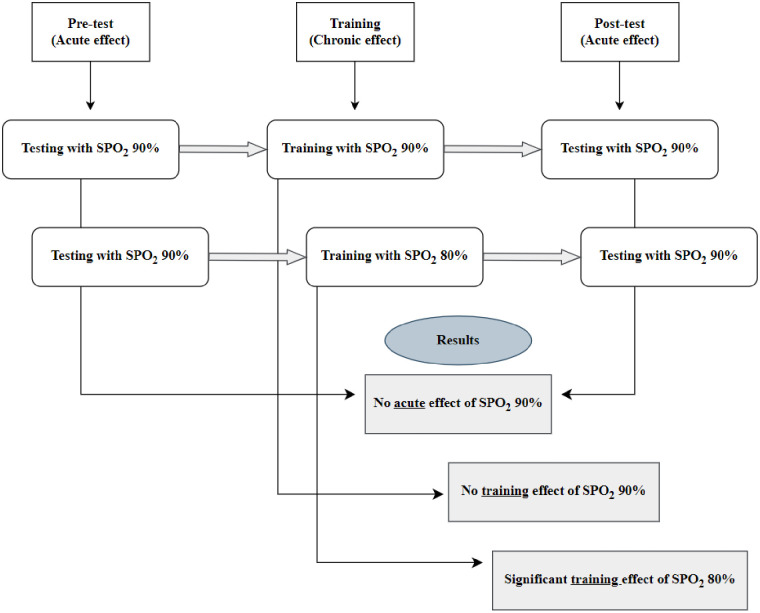
Representation of S_P_O_2_ levels for hypoxia training groups and hypoxic testing conditions.

Representation of S_P_O_2_ levels for hypoxia training groups and hypoxic testing conditions are given, and their results are summarized in Fig 6.

## Discussion

Our results showed that normobaric hypoxia has different effects on PAPE response when applied acutely or chronically. Regarding our first aim, when examining the acute effect, we found PAPE is unlikely to occur with DJ exercises under a hypoxic condition, but there is a significant PAPE response under the normoxic condition. Our second aim was to investigate the effect of training under hypoxic conditions on the PAPE response, and we found that performance increased at the 2^nd^ and 4^th^ minutes after a conditioning activity in the hypoxic group compared to the normoxic group, indicating a beneficial effect of hypoxic training.

Our acute-exercise effect results showed that jump height significantly increased in the 2^nd^ (31.7 ± 4.9) and 4^th^ (31.6 ± 4.3) minutes compared to baseline (30.1 ± 4.7) only after normoxic CA, and not hypoxic CA. These enhancements are also clinically worthwhile (SWC = 0.94) in our study, and should be considered practically useful performance improvements [37].

Mechanisms suggested for muscle improvement with hypoxia include increased vasodilation due to limited oxygen availability in hypoxia, and this vasodilation may contribute positively to the following exercise performance by increasing body temperature [3]. In our study, though we did not measure body temperature, we observed no improved subsequent exercise performance after hypoxic CA. In the study of Takei, Kakehata [38], sprinting under hypoxia followed by recovery under normoxia supplied increased physiological stimulus compared to sprinting in normoxia, and it was reported that this may provide greater oxygen availability during recovery, potentially affecting the following exercise performance. Also, these authors observed a decreased HR in the hypoxia condition compared to normoxia and reported that the reduction in HR may be due to compensatory vasodilation [38]. However, in our study, the acute hypoxic condition did not cause a positive effect on the following exercise performance in recovery. Although there was a significant difference in S_P_O_2_ levels between conditions after conditioning activity DJs, there was no difference in HR between conditions. Therefore, this may be related to the presence of any potential compensatory vasodilation induced by hypoxia due to the potential for dilation of arteries and arterioles [38], or our hypoxia condition may not have increased the exercise intensity enough to produce further performance improvement. The lack of a difference in our results of perceived exertion rate between the conditions may support the latter. However, our HR results are similar to the study of Scott, Slattery [13], which found no difference in HR between hypoxia and normoxia conditions and concluded that this may be associated with hypoxia level, which is moderate hypoxia (FIO_2 _= 16%) and appeared to be close to our S_P_O_2_ levels. In another study by Scott, Slattery [39], the results of RPE, which were not different between hypoxia and normoxia conditions during high-intensity resistance exercise, were also similar to our RPE results. RPE, which is an important indicator of exercise intensity [40], is similar between the two conditions, like HR in our study, too.

In the study on systemic hypoxia, the authors considered vasodilation and increased muscle blood flow at the beginning of the following exercise as a reason for performance enhancement in the subsequent exercise. In their study, the possibility of a higher baseline VO_2_ at the start of the following exercise was supported by lower blood lactate concentration values and higher basal VO_2_ levels found after a re-warm-up performed in hypoxia compared to normoxia [17]. However, conversely, some studies found higher blood lactate concentrations following resistance exercise in hypoxia compared to normoxia [13,41]. Ramos-Campo, Malta [17] attributed the increased CMJ and sprint performance observed after re-warm-up in hypoxia to post-activation potentiation, by associating that hypoxia inducement with higher motor unit recruitment. However, hypoxic exercise may be fiber-type selective, mostly influential on type II muscle fibers, and type II muscle fiber recruitment is related to increased exercise intensities [40,42], but regarding exercise intensity, we only have HR (Fig 4) and RPE (Table 2) results, which are similar between conditions. On the other hand, because of the possible effects on the central nervous system, severe hypoxia may impact motor unit recruitment [40], but our hypoxia level to test the acute effects (S_P_O_2_ 90%) is not severe. To be able to explain why acute hypoxia did not contribute to performance improvement in subsequent exercise in our study, we need to do further testing and know the changes in body temperature, blood lactate, muscle oxygenation, and muscle activation.

In our study, not only in pre-tests but also in post-tests after the 8-week training period, again, only normoxic CA resulted in significant PAPE in the 2^nd^ and 4^th^ minutes (Fig 3). There is no contribution of acute hypoxia to PAPE response even after chronic training adaptation.

Especially in high hypoxia (S_P_O_2_ < 75), decreases may occur in exercise performance [43]; however, fatigue accumulation during the training presents an extra effect that can affect the adaptations of the following training [44]. Therefore, although we did not find a positive effect of acute hypoxia (S_P_O_2_ 90%) on PAPE in both pre- and post-training, we found significant PAPE after an adaptation of 8 weeks of high hypoxia training (S_P_O_2_ 75%) (Table 3). In Table 3, pre-test PAPE results are seen as different at baseline, and the High-NH group showed a significant PAPE response already at the pre-test stage. Actually, at the beginning, we started with 8 participants for each training group by considering their pre-test results to make an equal group distribution. However, we could complete the study with some loss of participants, particularly from the High-NH group and the NN group. Due to this data loss, the baseline results varied between the groups, but our findings are still in favor of the High-NH group. The PAPE response, which was significant at the pre-tests, became insignificant when applied with 5 DJs at the post-test. This evidence indicates that the 8-week training was effective for only the High-NH group. When we applied the same test with more repetitions (with 8 DJs), we observed a significant PAPE response again, as shown in Table 3. This result may indicate that the group found the same number of repetitions (5 DJs) too light to elicit a PAPE response at the end of 8 weeks of training. Normally, the human body’s adaptive mechanisms do not react unless they are consistently asked to use more force to satisfy increased physiological demands [34]. However, despite 8 weeks of training, neither the test protocol with 5 DJs nor the test protocol with 8 DJs elicited a significant PAPE response in the other two groups. Moreover, even though the High-NH group did not show a significant PAPE response at the 2^nd^ minute in the pre-tests, it showed significant PAPE at both the 2^nd^ and 4^th^ minutes after the training period, showing that 8 weeks of plyometric training under high hypoxia helps PAPE appear earlier and that this response can last at least as long as it did in the pre-tests.

Amann, Goodall [45] found that acclimatization to high altitude mitigates the effect of acute hypoxia on the growth of central fatigue, whereas it does not mitigate the increase of exercise-related peripheral fatigue in acute hypoxia. Therefore, we thought factors related to peripheral fatigue may be a possible reason for impairment in the PAPE affected by acute hypoxia in our study. Central fatigue might have been attenuated with training (8 weeks), so we found PAPE in the High-NH group. However, even after adaptation by chronic hypoxic training (without group difference), there is still an impairment of PAPE again following hypoxic CA (Fig 3). Especially hypoxia level equal to FIO_2_ 0.13, reported with different S_P_O_2_ levels in previous studies (FIO_2_ = 0.13, S_P_O_2_ 83.7%) [46], (FIO_2 _= 0.13, SpO_2_ 76%) [47], may increase peripheral fatigue, some of which is similar to the S_P_O_2_ values that we observed with the DJs applied under acute hypoxia conditions in CA (S_P_O_2_ 85%) in our study. Exercise in hypoxia increases the stimulation of the anaerobic metabolic pathway [17], and it is known that anaerobic metabolism supports some processes of peripheral muscle fatigue [46].

Chronic hypoxia may provide adaptation in lowering the amount of central fatigue [48]. In our study, after adaptation to the 8-week High-NH training, we found improved PAPE responses, which also exceeded the smallest worthwhile effect for pre-training (SWC = 1.0 cm) and post-training (SWC = 0.9 cm) DJ performance. In the pre-test, it was a nonsignificant enhancement with 0.6 cm higher, and a significant improvement with 1.8 cm higher than the SWC in the 2^nd^ and 4^th^ min, respectively. In the post-tests (with 8 reps), significant enhancements were 1.2 cm higher in the 2^nd^ minute and 2.0 cm higher in the 4^th^ minute than the SWC.

There is no long-term study investigating the training effects of jump exercises on PAPE response, which is acute performance improvement, but a long-term study of hypoxic jump exercises is available to show chronic-training-induced jump performance improvements [6]. Coşkun, Aras [6] found greater jump performance enhancements after an 8-week jump exercise hypoxic training than the normoxic one. The authors were suspicious of higher muscle fiber recruitment as a potential reason for their further improvement by hypoxic training [6]. This was also proposed as a potential mechanism for the PAPE effect in the literature [17]. Hypoxic training leads to an earlier and greater type II fiber recruitment due to a decrease in oxygen concentration compared to normoxic training and thus may generate hypertrophic adaptation in these fibres as they are required to complete more work [42]. Regarding PAPE, the PAPE response is known to be higher in type II fibers. Rises in intracellular water, which can potentially be a reason for PAPE, may improve muscle force output. This effect is also higher in type II fibers [12]. Therefore, we can hypothesize that hypertrophy of type II fibers or higher fiber recruitment [6,42] may explain our training-effect results found in the High-NH group since severe hypoxia may impact motor unit recruitment by affecting the central nervous system [40]. On the other hand, despite the metabolite accumulation induced by resistance exercise in hypoxia, a substantial hypoxic dose may not be found at the muscular level. This was attributed to the moderate level of hypoxia (FIO_2_ = 16%) used in the previous study [13]. Bowtell, Cooke [1] investigated different inspired oxygen fractions (FIO_2_: 12%, 13%, 14%, 15%, 21%) and found gradually increased physiological responses as FIO_2_ dropped to 13%, but found significantly increased fatigue only at 12% FIO_2_ compared to normoxia. It is reported that locomotor exercise under acute hypoxia heightens peripheral fatigue, and chronic hypoxia may mitigate central fatigue. As the hypoxia intensity rises, the dominant factor changes to the central nervous system from peripheral fatigue mechanisms [48]. Therefore, the dose of hypoxia may affect different fatigue mechanisms (central/peripheral), which may explain the reason why we found different effects of acute and chronic hypoxia in this study.

Although we used two different hypoxic doses (80% and 90% S_P_O_2_) to test the long-term training effect of hypoxia, we tested the effect of an acute hypoxic stimulant with only a 90% S_P_O_2_ level to see the acute effects of hypoxic CA (Fig 6). We recommend examining both hypoxia levels as testing conditions (during CA), in future studies.

For future research, we recommend observing some other mechanistic physical and/or physiological testing, such as muscle temperature, blood lactate, muscle oxygenation, and muscle activation, and conducting the research on a higher number of participants. We determined the performance changes; however, physiological measurements are needed to understand the main mechanisms behind these performance results. The primary limitations were participant dropout and the absence of physiological tests. Although the groups were initially balanced in terms of sport branch and gender, participant dropout created variability in athletes’ schedules across the three groups.

In conclusion, plyometric exercise applied under normobaric hypoxia has different effects on PAPE response, acutely and chronically. While plyometrics with acute hypoxic CA with an S_P_O_2_ level of 90% has no beneficial effect on PAPE response, 8 weeks of plyometric training with normobaric hypoxia may lead to an adaptation to induce an improved PAPE. High hypoxia long-term DJ training with an S_P_O_2_ level of 80% substantially increased PAPE, while low hypoxia long-term training with an S_P_O_2_ level of 90% showed no benefit. According to our results, we can recommend that team-sport athletes add 8-week high-hypoxic DJ jump training into their training schedule during preparation for an important competition to have a PAPE advantage during competitions.

## Supporting information

S1 File PAPE data. (XLSX)
